# Establishment of a ^123^I-*meta*-iodobenzylguanidine normal database for D-SPECT using Japanese and Italian data

**DOI:** 10.1007/s12149-025-02061-4

**Published:** 2025-06-02

**Authors:** Xue Zhang, Kenichi Nakajima, Wanda Acampa, Roberta Assante, Adriana D’Antonio, Koichi Okuda, Kunihiko Yokoyama, Seigo Kinuya

**Affiliations:** 1 Department of Nuclear Medicine, Kanazawa University, 13-1 Takara-machi, Kanazawa, 920-8640 Japan; 2 Department of Functional Imaging and Artificial Intelligence, Kanazawa University, Kanazawa, Japan; 3 Department of Advanced Biomedical Science, University Federico II of Naples, Naples, Italy; 4 Department of Radiological Technology, Hirosaki University School of Health Sciences, Hirosaki, Japan; 5 Public Central Hospital of Matto Ishikawa, Hakusan, Japan

**Keywords:** Sympathetic imaging, Defect score, Coronary artery disease, Cardiomyopathy

## Abstract

**Purpose:**

We aimed to develop ^123^I-*meta*-iodobenzylguanidine (MIBG) normal databases (NDBs) for D-SPECT that included Japanese and European populations and to validate the ability of combined NDBs to diagnose heart failure (HF).

**Methods:**

This study included 55 Japanese and 33 Italian patients without cardiac or neurological diseases associated with Lewy bodies. Single-photon emission computed tomography (SPECT) images were acquired 20 min and 3–4 h after ^123^I-MIBG injection. We established NDBs for Japanese (Jp-NDB) and Italian (It-NDB) patients, and combined them (JpIt-NDB) using 17-segment polar maps. We also compared the Jp- with the Anger camera NDB established by the Japanese Society of Nuclear Medicine and clinically validated our findings in a cohort of 31 patients with HF.

**Results:**

The average uptake in the anterior segments was lower in the It-NDB and differences were more pronounced in males. The JpIt-NDB closely aligned with both the Jp-NDB and It-NDB. Clinical validation indicated that HF was identified more accurately by automated defect scores derived from the JpIt-NDB than visual scores (area under the receiver operating characteristic (ROC) curve (AUC): 0.827 *vs*. 0.683; *P* = 0.032). Combining JpIt-NDB-derived scores based on the heart-to-mediastinum ratio (HMR) significantly enhanced diagnostic accuracy (early and late AUCs: 0.960 and 0.952, respectively).

**Conclusions:**

The combined JpIt-NDB accurately diagnosed HF in Japanese and European patients, while the defect scores correlated closely to Jp- and It-NDBs. Integrated HMR and NDB defect scores significantly enhanced cardiac assessment precision, which offers promise for future investigations into cardiac innervation imaging.

## Introduction

Cardiac scintigraphy with the norepinephrine analog ^123^I-meta-iodo-benzyl-guanidine (MIBG) has become an established method of assessing sympathetic neuronal function in the heart [1]. The heart-to-mediastinum average count ratio (HMR) derived from planar images is the most prevalent semi-quantitative parameter with which to reflect ^123^I-MIBG uptake. A lower HMR correlates with a poorer prognosis of heart failure (HF), regardless of left ventricular ejection fraction (LVEF) and New York Heart Association (NYHA) clinical conditions [2–5].

Despite having a comprehensive perspective, planar imaging has several limitations. It cannot provide regional analysis and overlapping non-cardiac structures, and different camera collimators can result in inconsistent outcomes [6]. To address these drawbacks of planar images, ^123^I-MIBG SPECT is applied as a complement. Degrees of segmental defects are calculated from a 17-segment polar map, which leads to more accurate determination of regional denervation and quantitation of cardiac neuronal dysfunction [7].

Despite its benefits, heterogeneous uptake can manifest as decreased activity in the inferior wall or apex, particularly among older persons. Moreover, uptake in surrounding organs such as the lungs and liver can affect cardiac uptake, increasing the likelihood of false-positive and −negative results when segmental defects are visually scored [8–10]. The automated quantitation provided by QPS software (Cedars Sinai Medical Center, Los Angeles, CA, USA) is a valuable tool for assessing severity and the extent of defects. However, accurate automated quantitation requires a standardized normal database.

A dedicated cardiac SPECT camera (D-SPECT) has been developed for myocardial imaging using cadmium–zinc telluride (CZT) detectors (Spectrum Dynamic Medical, Caesarea, Israel). It has improved spatial and energy resolution and higher sensitivity, providing better MIBG image quality compared with conventional Anger camera. This has been validated by myocardial perfusion (MPI) and myocardial adrenergic functional imaging [11–13].

The Japanese Society of Nuclear Medicine (JSNM) working group created a ^123^I-MIBG normal database using data from conventional Anger SPECT images of Japanese patients [8, 14], which have been used for clinical studies to quantitate locations and severity of defective segments. However, a ^123^I-MIBG normal database adjusted for D-SPECT has not yet been established. Moreover, physiological and body stature differences between Asian and Caucasian populations highlight the need for a population-specific or appropriate universal database. We aimed to establish individual and combined ^123^I-MIBG D-SPECT normal databases for ^123^I-MIBG D-SPECT derived from information about Japanese and Italian patients and to validate them for clinical use in patients with HF.

## Methods

### Patients

This study included 55 Japanese patients (male, *n* = 28; female, *n* = 27) from Kanazawa University Hospital and 33 Italian patients (male, *n* = 13; female, *n* = 20) from the University Federico II of Naples, who were assessed using early and late ^123^I-MIBG D-SPECT images. Table 1 shows the patient characteristics and Table 2 shows the inclusion criteria. Japanese patients without moderate or severe cardiac disease, dementia with Lewy bodies (DLB), or Parkinson’s disease (PD) were selected from Neurology and Cardiology departments. These patients were assessed by planar ^123^I-MIBG scans using an Anger camera, to generate a basis for comparison with D-SPECT data. The Italian patients were referred for adrenal evaluation and were negative for pheochromocytoma. Anterior images were derived from planograms created during D-SPECT image processing.

**Table 1:** Characteristics of populations included in the creation of the normal database

	Japanese (*n* = 55)	Italian (*n* = 33)	*P*
Age (y)	75.1 ± 9.41	50.8 ± 12.83	< 0.0001
Female	27 (49%)	20 (61%)	NS
BMI	21.8 ± 4.53	26.5 ± 4.62	< 0.0001
Diabetes	13 (23.6%)	4 (12%)	NS
Hypertension	18 (32.7%)	21 (64%)	0.005
Hyperlipidemia	11 (20%)	7 (21%)	NS
Chronic kidney disease	10 (18.2%)	NA	NA
LVEF (%)	66.3 ± 5.55	63.4 ± 4.3	NS

Data are shown as means ± SD or n (%) unless otherwise specified

*BMI* body mass index, *SD* standard deviation, *LVEF* left ventricular ejection fraction, *NA* not available, *NS* not significant

**Table 2:** Inclusion criteria for the normal database

NDB	Criteria
Japanese	
	Neurology
	Neither DLB nor PD
	No history of cardiac disease
	No severe focal defects on perfusion imaging
	No symptoms suggesting CAD or HF
	Follow-up > 2 years and confirmed cardiac complication free
	Anger camera MIBG
	Cardiology
	Coronary artery stenosis < 50%
	LVEF > 50%, no LV dilation
	NYHA class I acceptable
	Indication for PAF ablation acceptable
	Normal ECG
	No CKD
	No DLB signs
	Anger camera MIBG
Italian	
	Oncology
	No history or symptoms of CAD
	No history or symptoms of neurological disease
	Normal ECG
	Normal clinical evaluations
	D-SPECT acquisition

*CAD* coronary artery disease, *CKD* chronic kidney disease, *DLB* dementia with Lewy bodies, *ECG* electrocardiography, *HF* heart failure, *LV* left ventricle, *LVEF* LV ejection fraction, *MIBG meta*-iodobenzylguanidine, *PAF* paroxysmal atrial fibrillation, *PD* Parkinson disease

The ethics committee at Kanazawa University approved the study protocol (Approval number: 3266/2019-244) and all participating institutions approved the multicenter data collection. The requirement for written informed consent was waived given the retrospective nature of the study. All image data were transferred to a core laboratory in the anonymized form.

### Data acquisition and establishing of NDBs

Early and late images were acquired at 15–20 min and 3–4 h after patients were injected with 111 MBq of ^123^I-MIBG (MyoMIBG; PDRadiopharma Inc., Tokyo, Japan) and 185 MBq of AdreView (GE Healthcare, Milan, Italy). Data were collected in Japan using an Anger camera (Siemens Healthcare, Tokyo, Japan), followed immediately by D-SPECT image acquisition. Conventional planar images of supine patients were acquired using the Anger camera for 5 min in a 256 × 256 matrix with a low-energy high-resolution (LEHR) collimator. The D-SPECT data in Japan and Italy were acquired using single-nuclide ^123^I-MIBG. The acquisition energy was centered at 159 keV with a 15% window (−7.5% to + 7.5%) for both NDBs. A Japanese validation dataset was generated during a single-nuclide ^123^I-MIBG study. An Italian validation dataset was generated from images of patients with HF acquired using 74 and 185 MBq of ^123^I-MIBG/^99 m^Tc-sestamibi, respectively. Asymmetrical windows of 8.5% (−7.5–4.5 keV) and 10% (−7% to + 9%) were centered at 159 and 140.5 keV for ^123^I and ^99 m^Tc, respectively. Cross talk from the ^99 m^Tc to ^123^I energy window was < 1%. SPECT images of seated patients were acquired for 10 min using a system with nine mobile CZT detectors, each providing one elementary two-dimensional (2D) image per angle. The original data were anonymously transferred to the Digital Imaging and Communications in Medicine (DICOM) format and collected at Kanazawa University. The data were then incorporated into the QPS software algorithm using the normal limits generator to create 17-segment polar maps, which characterized the means and mean deviation for each segment. Based on this, one NDB each was established for the Japanese (Jp-), Italian (It-), and combined (JpIt-) NDBs. The HMR was also calculated using smartMIBG software (PDRadiopharma Inc.) with data derived using an Anger camera in Japan [15, 16]. The HMR was calculated from planogram-based cardiac and mediastinal ROIs using H2M software (Spectrum Dynamics Medical) in Italy. The planogram-based and Anger camera-based HMRs were standardized to a medium-energy general-purpose collimator condition [17].

A normal database for the Anger camera was derived from the Japanese Society of Nuclear Medicine working group (JSNM-WG) database, which is currently used for clinical practice in Japan [8, 18]. We used an early and late NDB for 180° SPECT data acquisition.

### Clinical validation

A validation study was conducted to verify the performance of the ^123^I-MIBG D-SPECT NDB. Data were obtained from 43 patients, among whom 31 had HF (male, *n* = 23; female, *n* = 8). The diagnosis of HF was made by cardiologists based on a comprehensive clinical assessment, including symptoms, echocardiographic findings, blood test results such as b-type natriuretic peptides (BNP) or N-terminal-proBNP, and clinical course. A total of 19 were diagnosed with CAD and 8 with dilated cardiomyopathy (DCM). We also analyzed data from 12 (male, *n* = 9; female, *n* = 3) persons with a low likelihood of cardiac diseases from Italy and Japan (controls). Patients with neurological disorders were excluded due to significant uptake deficits.

Images were processed and data acquired in the same way that the NDB  was constructed. Cardiac sympathetic innervation was assessed using the standard 17-segment model by scoring uptake described as 0 (normal), 1 (slight), 2 (moderate), 3 (severely decreased), and 4 (absent). The summed defect scores were calculated as summed early (SES) and late (SLS) scores. The QPS algorithm provided automatic scoring, whereas visual scores were judged by three experts. Visual scores of 0, 1, 2, 3, and 4 were essentially based on %counts/segment and were defined as ≥ 70%, 60–69%, 50–59%, 40–49%, and < 40%, respectively.

Correlations and differences between automated defect scores derived from the JpIt-NDB and those from the Jp- and It-NDBs were analyzed to determine the applicability of the JpIt-NDB to diverse populations. Relationships between JpIt-NDB automated and visual defect scores and HMR were assessed, and we compared the JpIt-NDB automated defect scores among patients with CAD or DCM, or neither. The diagnostic quality of JpIt-NDB automated defect scores was evaluated alone and combined with the HMR, and compared with visual defect scores.

### Statistics

Continuous and categorical variables are expressed as means ± standard deviation (SD) and ratios (%), respectively. Segmental differences among NDBs were assessed using one-way analysis of variance (ANOVA), with mean deviations converted to SDs assuming a normal distribution. Relationships between scoring methods were analyzed using Pearson linear correlations and Bland–Altman plots. The HMR and JpIt-NDB automated defect scores were compared among different diseases using Kruskal–Wallis test, and diagnostic accuracy was evaluated using receiver-operating characteristics (ROC) curves. Multivariable logistic model was used to combine the HMR and defect scores. Data were statistically analyzed using JMP pro 17.2 (SAS Institute, Cary, NC, USA). Values with P < 0.05 were considered statistically significant.

## Results

### Comparison of NDBs

**Fig. 1.**
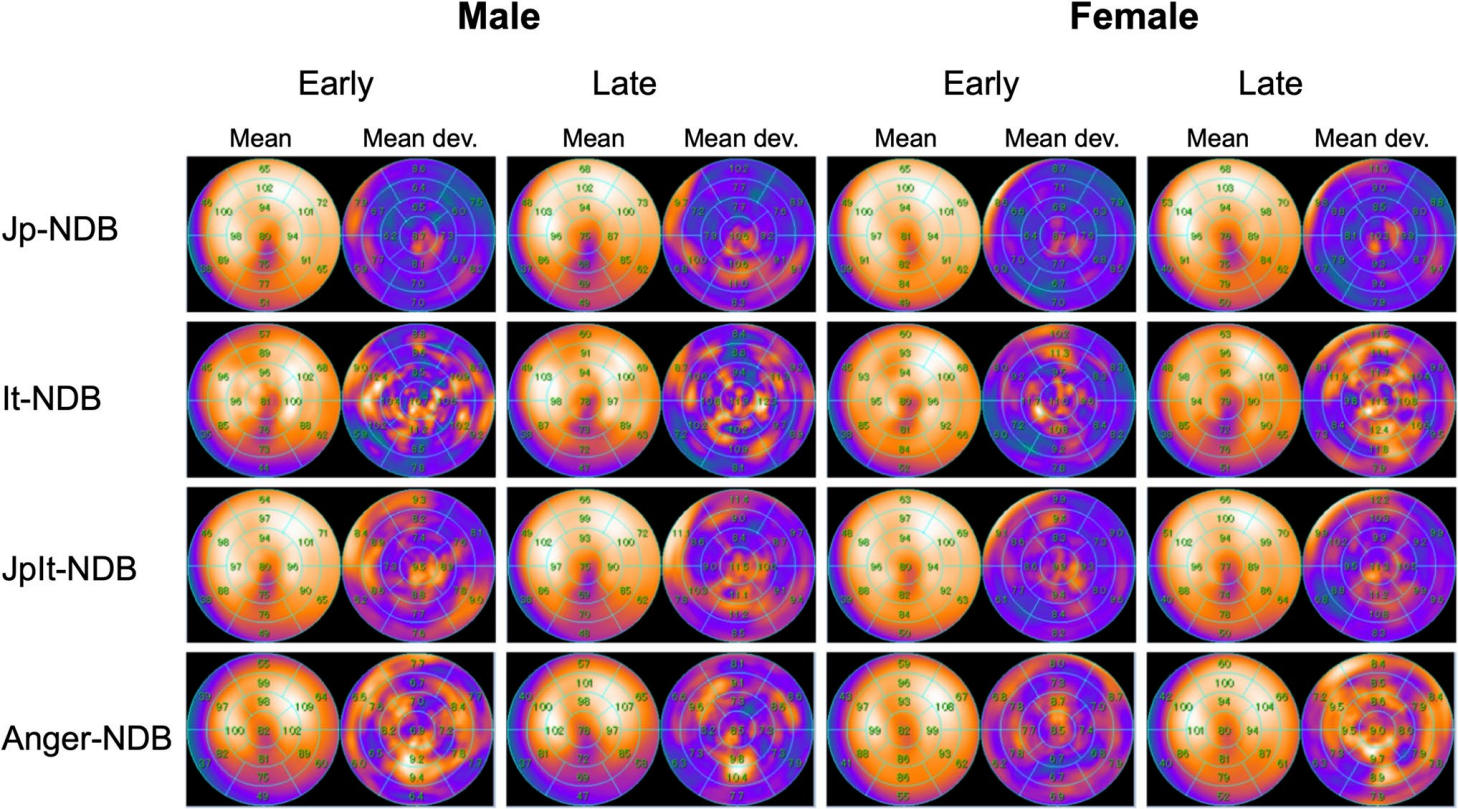
^123^I-MIBG normal Japanese (Jp-NDB), Italian (It-NDB), combined population (JpIt-NDB) and Anger camera (Anger-NDB) databases

**Fig. 2:**
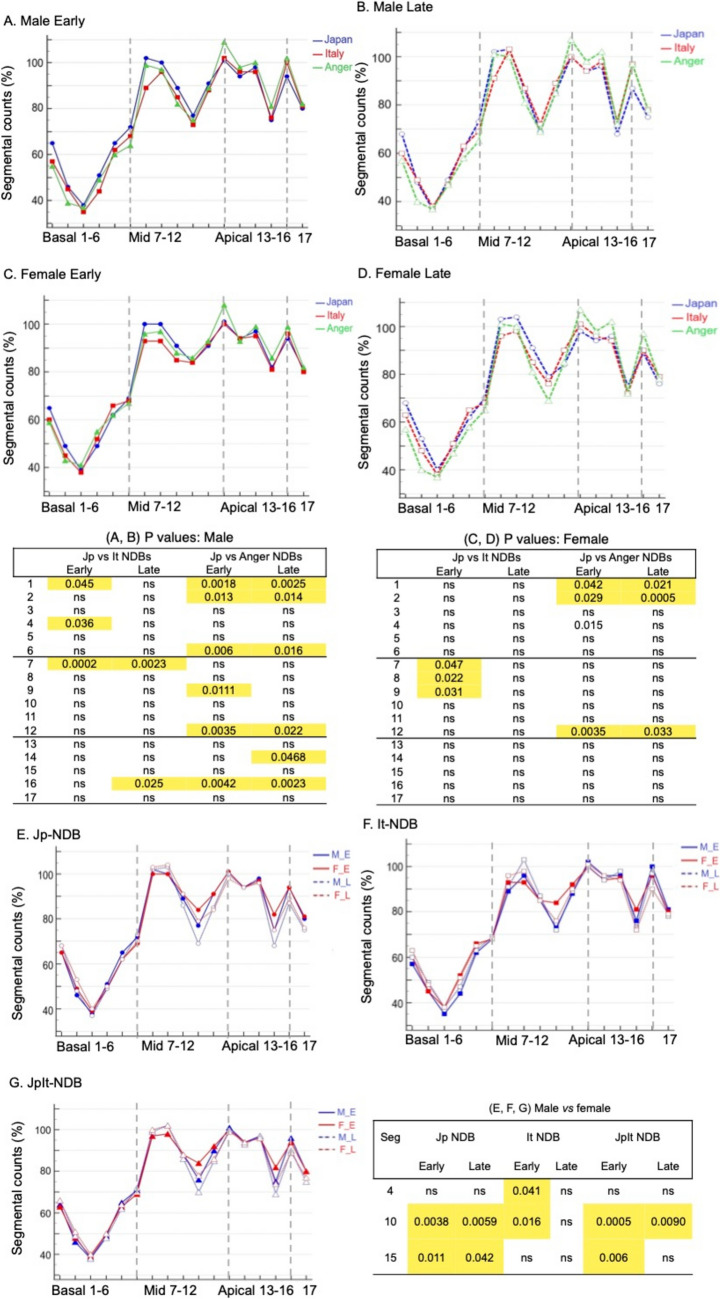
Detailed comparison of segmental values for males between Jp-NDB normal database (NDB), It-NDB, and Anger-NDB (**A**, **B**) and females (**C**, **D**); sex-specific comparison of segmental values in Jp-NDB (**E**), It-NDB (**F**), and JpIt-NDB (**G**). Segments with insignificant P values are not shown in panels (**E**, **F**, and **G**)

Figure 1 shows the established NDBs. Figure 2A–D shows the segmental values for males and females among the NDBs. Values in males were respectively higher and lower in apical and basal segments when generated by the traditional Anger camera, than the D-SPECT Jp-NDB in early and late images. Only basal segments 1, 2, and 6, and mid-anterolateral and apical lateral segments 12 and 16, respectively, significantly differed (*P* < 0.05; Fig. 2A, B). This profile was also evident in females, although it was less prominent, with significant variations in basal segments 1 and 2, and mid-anterolateral segment 12 (*P* < 0.05; Fig. 2C, D).

More discrepancies between Italian and Japanese male patients were evident in early images. The It-NDB generated notably lower values in the mid-anterior segments in early and late images, and in basal-anterior and -inferior segments in early images. The It-NDB generated higher values in the apical-lateral area at the late stage, but large deviations might have led to inaccuracies (Fig. 2A, B). Values in females in late images did not significantly differ between the Jp-NDB and It-NDB, whereas differences were notable in the mid-anterior, -anteroseptal, and -inferoseptal segments in early images (*P* < 0.05; Fig. 2C, D).

Figure 2E–G compares the segmental values of three NDBs between males and females. Though to various extents, the values in the inferior segments were lower for males than females according to the Jp-NDB (significant in apical-inferior and mid-inferior regions in early and late images), the It-NDB (significant in basal-inferior and mid-inferior regions at the early stage), or the JpIt-NDB (significant in apical-inferior region at both stages and mid-inferior region in early images).

### Correlation and differences of automated defect scores among established NDBs

**Fig. 3:**
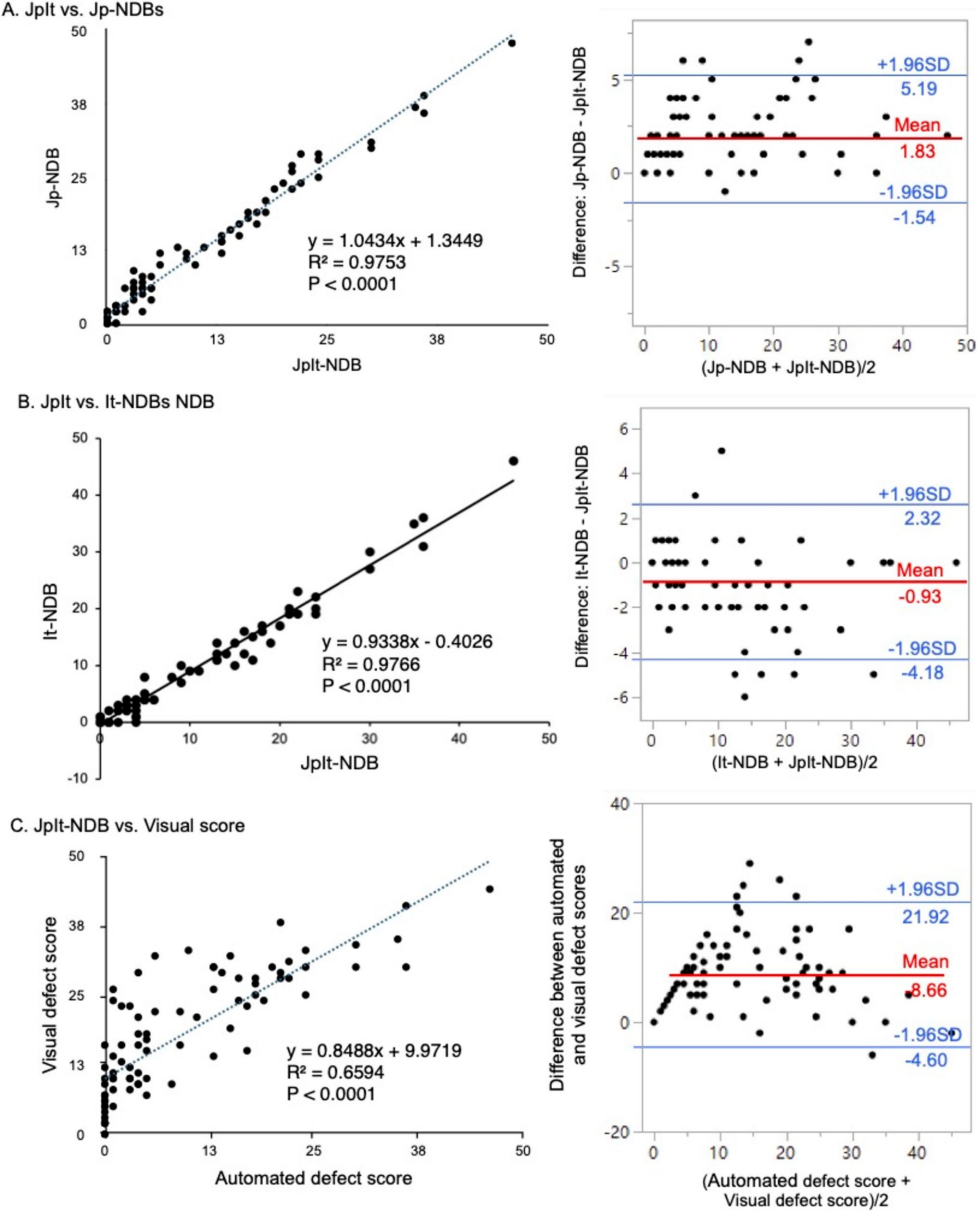
Correlations (left) and differences (right) in automated defect scores between **A** JpIt- and Jp-, **B** JpIt- and It- and **C** JpIt-NDB automated and visual defect scores

The results of the linear correlation and Bland–Altman plots indicated that the defect scores automatically calculated by the JpIt-, Jp-, and It-NDBs closely correlated (Fig. 3A, B). Specifically, the correlation coefficients of the automated defect scores between the JpIt- and Jp-NDBs and between the JpIt- and It-NDBs were 0.975 (mean difference, 1.83; 95% limits of agreement (LOA) −1.54–5.19) and 0.977 (mean difference, −0.93; 95% LOA −4.18–2.32 (*P* < 0.0001 for both).

**Fig. 4:**
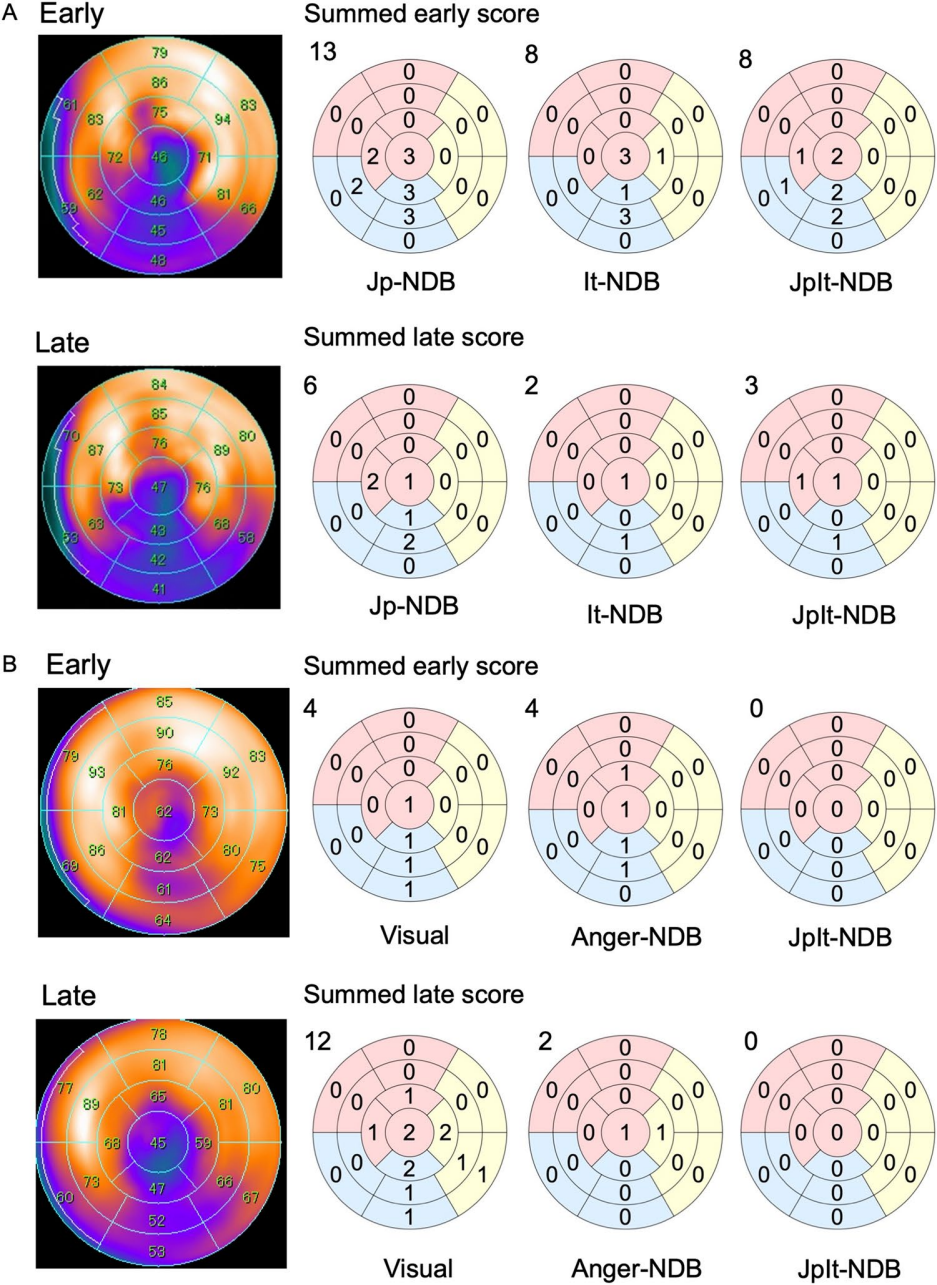
Examples of validation using polar maps. **A** Polar maps and automated defect scores for a 97-year-old female with chronic heart failure, generated by each NDB, **B** Polar maps and defect scores for an 80-year-old male with paroxysmal atrial fibrillation and multiple system atrophy, calculated from visual assessments, Anger-NDB, and JpIt-NDB

Figure 4A shows an example of polar maps and automated defect scores calculated by each NDB for a 97-year-old female with CHF. Despite the correlations, the automated defect scores differed among the NDBs. The calculated scores generated by the JpIt-NDB were higher than those of the It-NDB and lower than those of the Jp-NDB in early and late images. These results aligned with the Bland–Altman findings.

### Diagnostic efficacy of JpIt-NDB automated defect score

The analysis indicated significantly higher visual than automated defect scores (mean difference, 8.66; 95% LOA −4.60–21.92 (*P* < 0.0001). Although the correlation between the methods was linear, the overall fit was suboptimal (Fig. 3C).

Figure 4B shows an example of polar maps and defect scores calculated using visual assessment, Anger-, and JpIt-NDBs for an 80-year-old male with multiple system atrophy, chronic obstructive pulmonary disease, and paroxysmal atrial fibrillation. This emphasized significant differences in defect scores between the visual and automated calculations. The visual defect scores were the highest and primarily located in the apical and inferior regions, whereas the JpIt-NDB automated defect scores were 0 in early and late images.

**Fig. 5:**
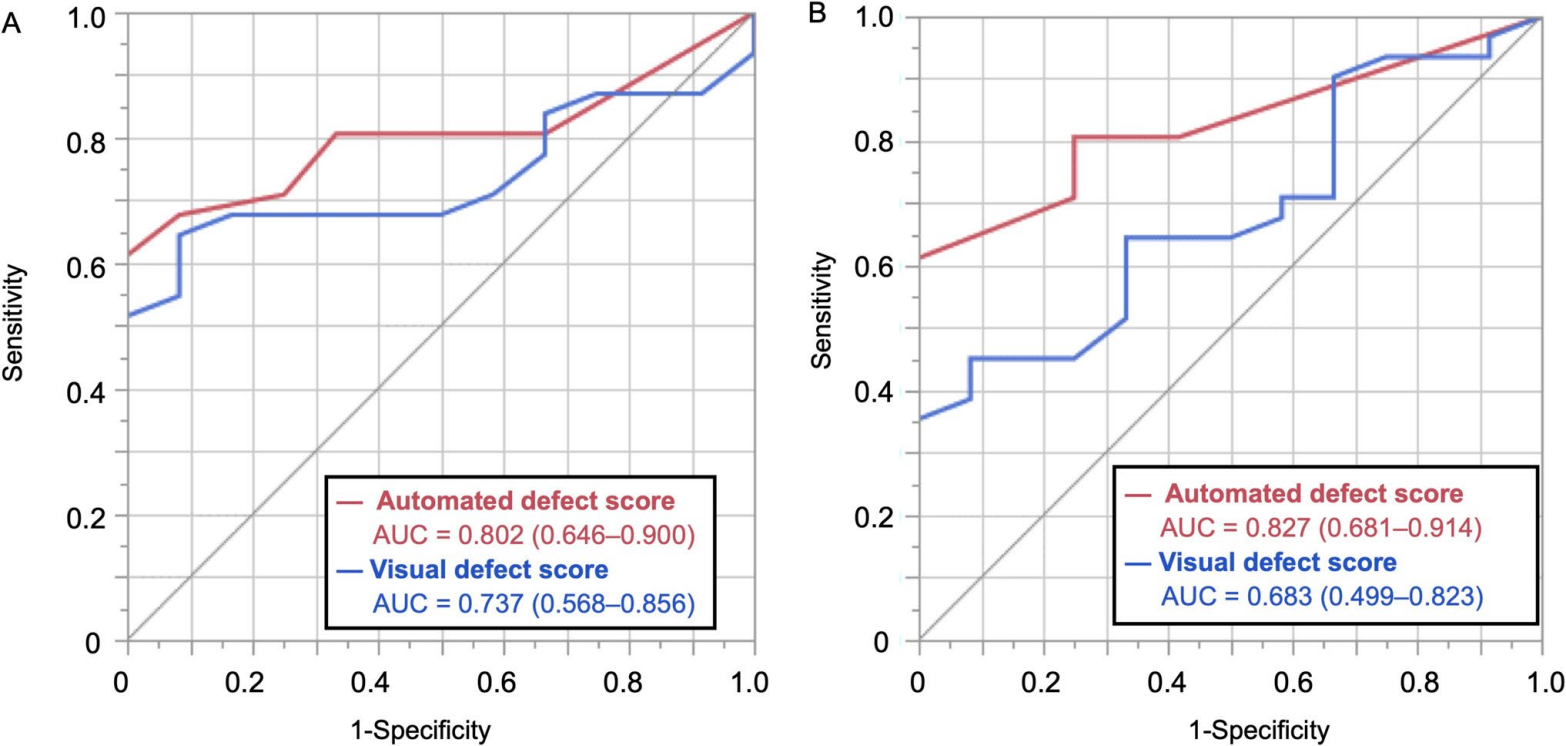
Comparison of ROC analysis of JpIt-NDB-based automated and visual defect scores in early (**A**) and late (**B**) images

We compared the ability of visual and JpIt-NDB automated defect scores to detect HF due to CAD and/or DCM using ROC curves (Fig. 5). The AUCs were 0.737 (95% CI 0.568–0.856) and 0.802 (0.646–0.900) for visual and JpIt-NDB automated defect scores, with no significant differences (*P* = 0.076). At the late stage, the JpIt-NDB automated defect scores were more effective than visual defect scores (*P* = 0.032), with AUCs of 0.827 (95%CI 0.681–0.914) and 0.683 (0.499–0.823), respectively. Sensitivity, specificity, and accuracy of visual defect scores of early and late uptake were, respectively, 65%, 92%, and 72%, and 45%, 92%, and 58%. These metrics determined by JpIt-NDB automated defect scores were 61%, 100%, and 72% during early and late uptake.

### Diagnostic efficacy of combining HMR with automated defect scores calculated by JpIt-NDB

**Fig. 6:**
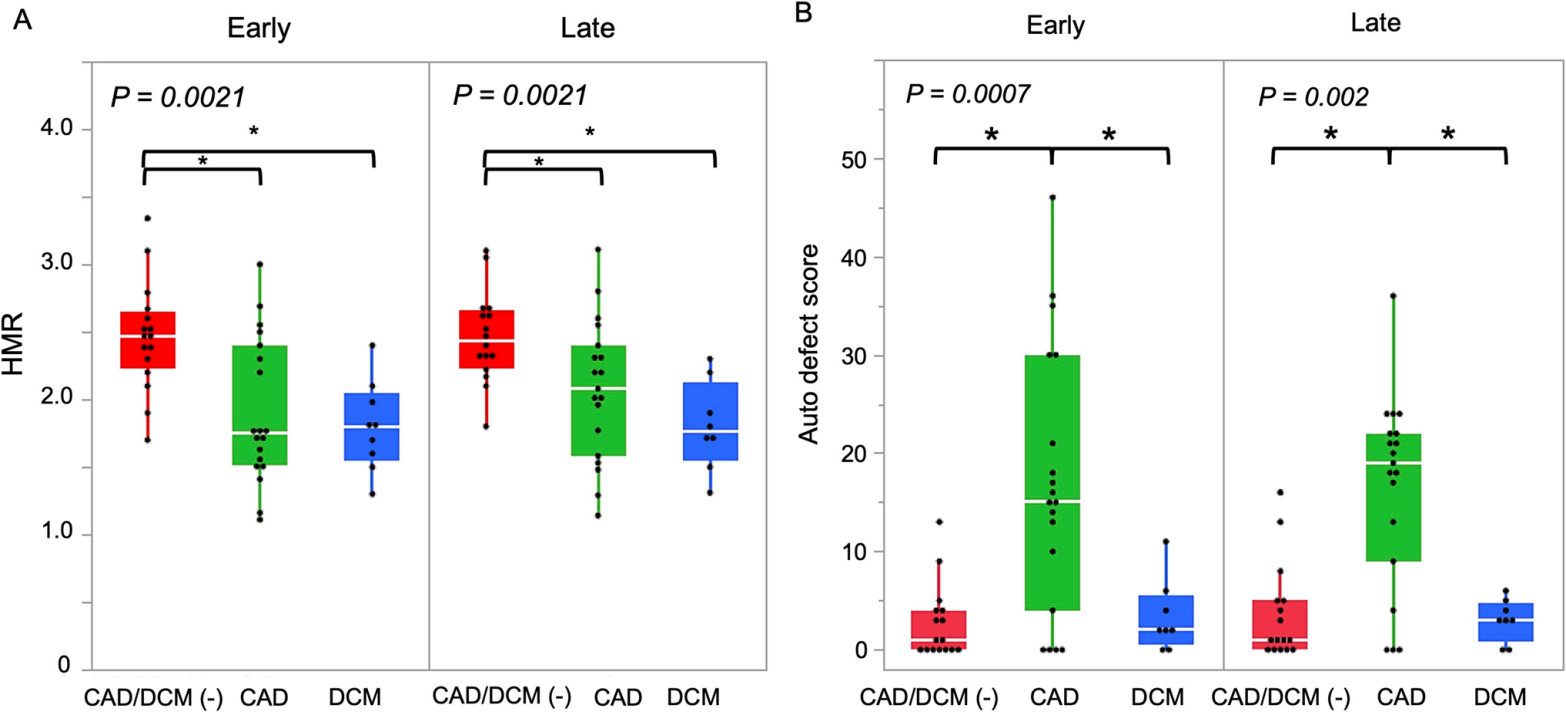
Comparison of heart-to-mediastinum ratio (HMR) (**A**) and automated defect scores calculated using JpIt-NDB (**B**) among patients with coronary artery disease (CAD), dilated cardiomyopathy (DCM), and neither CAD nor DCM. P values indicate the overall statistical significance among the three groups as determined by the Kruskal–Wallis test. *P < 0.05

The HMR and the automated defect scores generated by the JpIt-NDB significantly differed among the groups with CAD and DCM and without either disease (*P* < 0.05) (Fig. 6). The significantly different trends of both methods among patients with CAD or DCM were similar during early and late uptake.

The HMR values were significantly lower in patients with, than without either CAD or DCM (*P* < 0.05). Specifically, the mean early and late HMR values were 2.07, 1.91 for CAD, and 1.81 and 1.78 for DCM, and 2.46 and 2.47 for the group with without neither of these diseases, respectively.

The mean early and late defect scores calculated by the JpIt-NDB significantly increased in the CAD group (early and late: 16.4 and 16.8, respectively). In contrast, the mean defect scores for early and late uptake were similar between the group with DCM and that without either DCM or CAD (early, 3.0 and 3.6; late, 3.5 and 2.7, respectively). These results differed from the HMRs, which significantly differed between patients with CAD and DCM as well as the group without either CAD or DCM (*P* < 0.05).

**Fig. 7:**
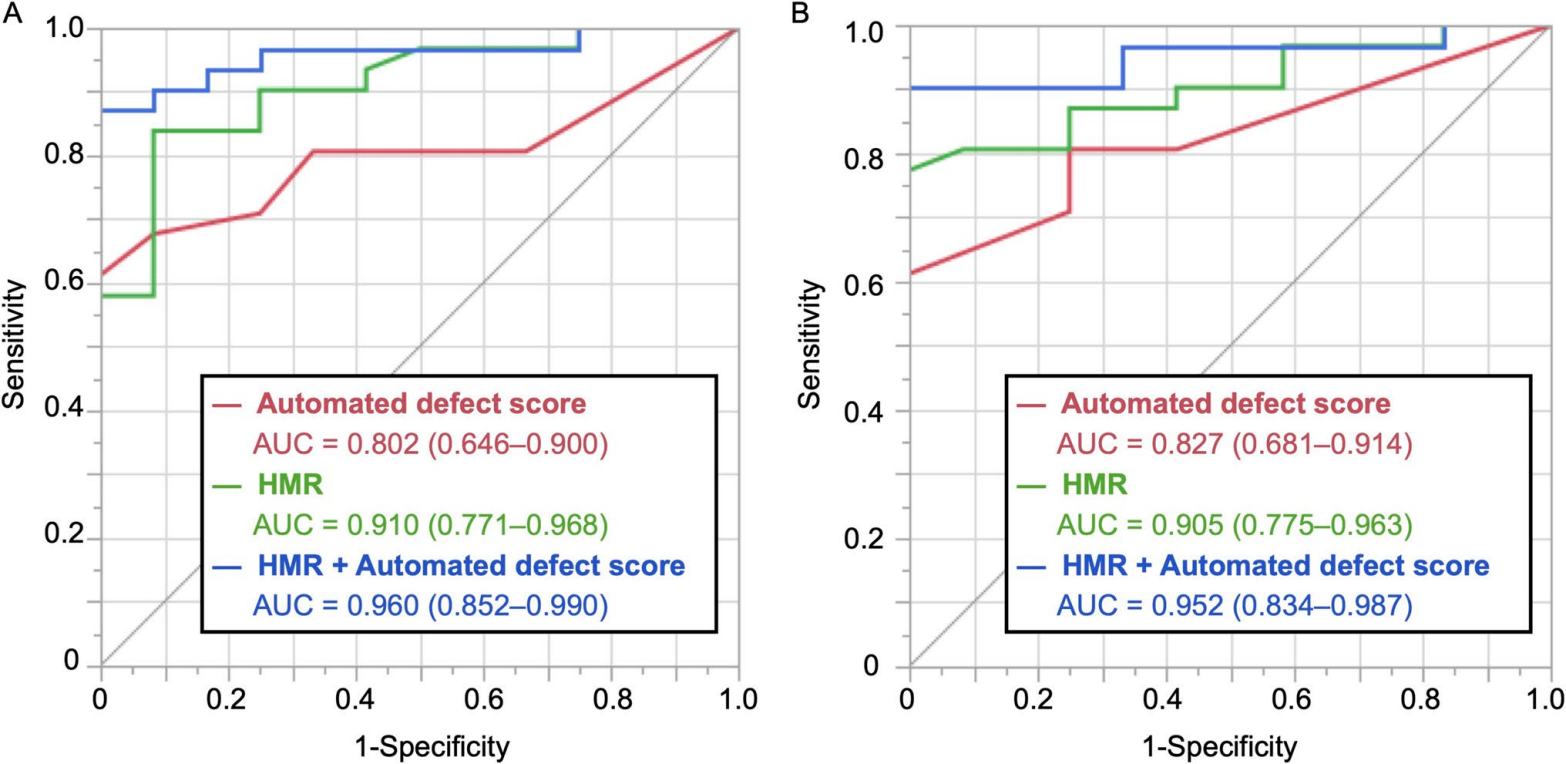
Comparison of ROC curves for JpIt-NDB automated defect scores and HMR alone and combined in early (**A**) and late (**B**) images

Due to variations among the HMRs and defect scores, we combined these two indexes to determine whether this would improve the sensitively of detecting HF (Fig. 7). The early and late AUCs were 0.960 (0.852–0.990) and 0.952 (0.834–0.987), respectively, both of which were higher than using either method (particularly, the automated defect score) alone (combination *vs.* HMR: early, *P* = 0.0086 and 0.0141 *vs.* *P* = 0.12; late, 0.15, respectively). The sensitivity of the combination was substantially increased compared with either alone, reaching 87% and 90% during early and late uptake, respectively.

## Discussion

In this study, we established ^123^I-MIBG NDBs specifically tailored for D-SPECT imaging and validated their diagnostic utility in patients with heart failure. The results demonstrated that these NDBs, including the combined Japanese and Italian NDB, enabled accurate defect scoring and improved diagnostic performance. Notably, SPECT-based defect scores and planar image-derived HMR values exhibited differing behaviors in patients with DCM and CAD, suggesting that the combined use of both metrics may enhance diagnostic accuracy.

## Need for ^123^I-MIBG D-SPECT normal databases

In Japan, cardiac ^123^I-MIBG studies routinely incorporate both planar and imaging to ensure comprehensive evaluation. Standardized quantitative assessment requires dedicated NDBs. While the JSNM-WG previously developed NDBs for Anger camera systems [8, 14], D-SPECT—with its CZT detectors offering superior count sensitivity and energy resolution—necessitates the creation of new modality-specific databases. Although D-SPECT NDBs have been established for myocardial perfusion imaging (MPI) [19–21], no equivalent ^123^I-MIBG NDB for D-SPECT has been available. To address this gap and enhance international applicability, we established ^123^I-MIBG D-SPECT NDBs using Japanese and Italian cohorts and created a combined database. The results confirmed the significant relevance of these NDBs and revealed that automated defect scores derived from the JpIt-NDB can diagnose HF more sensitively than traditional visual defect scores. Furthermore, combining the HMRs with automated defect scores conferred better diagnostic accuracy than either method alone, which warrants consideration in clinical practice.

## Diagnostic value of SPECT-based defect scores versus planar HMRs

Although planar HMR has demonstrated prognostic value in patients with HF [3, 5, 22], it lacks sensitivity for detecting regional sympathetic denervation, which is clinically relevant in conditions such as arrhythmia, vasospastic angina, and Takotsubo syndrome. SPECT-based defect scoring better identifies innervation–perfusion mismatch, which planar imaging can miss.

For instance, in the context of arrhythmia, regional disparities in myocardial sympathetic innervation contribute to an increased susceptibility to malignant arrhythmias in patients with ischemic heart failure [23]. This disparity is evident on ^123^I-MIBG SPECT imaging evaluated using the standard 17-segment model, with arrhythmic patients often exhibiting larger areas of innervation defects than perfusion defects [24–26]. Such localized mismatches may remain undetected when relying solely on planar ^123^I-MIBG imaging, potentially resulting in the omission of clinically significant abnormalities.

## Comparison of NDBs

The parameters of a single phase of ^123^I-MIBG imaging have been investigated in detail. While late-phase imaging is favored for mapping regional sympathetic denervation [27], early-phase imaging correlates more closely with impaired myocardial contractility [13, 28]. Thus, the choice of imaging phase apparently depends largely on specific research objectives and clinical needs. To accommodate these varying applications, we established and validated ^123^I-MIBG NDBs for early and late stages, which allowed selection of the most appropriate NDB for investigative purposes.

The uptake of ^123^I-MIBG in healthy individuals is influenced by factors such as race, sex, equipment, and other variables. We therefore compared the performance and characteristics of various NDBs.

Regardless of imaging phase or sex, D-SPECT consistently identified significantly higher uptake in the basal anterior, anteroseptal, and anterolateral regions, whereas the Anger camera revealed increased uptake in the apical-lateral and mid-anterolateral regions, particularly around the apex. These differences can be attributed to variations in examination positioning, different camera angles, and attenuation in deep regions according to myocardial perfusion imaging (MPI) findings [29, 30].

Uptake is notably lower in inferior segments of males than females according to all NDBs. An age-related decline in MIBG uptake in the inferior wall among males was identified, whereas this effect is less pronounced in females [31]. This discrepancy might be attributed to a decline in parasympathetic nerve activity in males and sympathetic modulation with aging; this phenomenon is less evident in females. Given that the inferior and anterior walls are predominantly innervated by parasympathetic and sympathetic nerves, respectively, these differences in autonomic regulation likely contribute to the reduced uptake in the inferior region among males [31, 32].

The Japanese and Italian NDBs primarily differed in terms of segments affected by extracardiac soft tissues, such as the diaphragm and breasts, which was consistent with previous findings [20, 33]. This variation is partly due to differences in body composition, with 24 of 33 Italian patients being classified as overweight or obese (BMI > 24), compared to 9 of 55 Japanese individuals. We speculate that the impact of BMI-related attenuation is primarily manifested in the inferior and anterior walls in males due to abdominal fat and chest musculature, and in the lateral wall in females due to breast tissue, which resulted in observed differences between the Japanese and Italian NDBs. Several studies have suggested that age may influence ^123^I-MIBG myocardial uptake and distribution. However, no consistent consensus has been reached, and such effects appear to be modulated by other factors [31, 32, 34, 35]. In our study, although the Japanese patients were older on average, no reduction in inferior wall uptake was observed compared to the Italian patients. This seemingly paradoxical finding might be influenced by a combination of factors, including BMI, ethnic or physiological differences in sympathetic nerve distribution or norepinephrine transporter expression, and variations in imaging protocols or radiotracer kinetics, which may have obscured potential age-related effects on ^123^I-MIBG uptake. Moreover, heterogeneity was greater for the It-, than the Jp-NDB, likely due to stricter selection criteria and higher genetic homogeneity among the Japanese population [36, 37]. These variations have led to discrepancies in defect score calculations, with higher and lower scores being generated in the Jp-NDB and It-NDB, respectively. Although the importance of population-specific NDBs has been emphasized [38], the need for accessibility and reproducibility clarifies the need for a more universally applicable database.

Therefore, we developed a combined JpIt-NDB using comparable acquisition protocols and standardization methods. Although age differences existed between the Japanese and Italian populations, clinical practice often requires more accessible and generalizable tools. In real-world settings, patients undergoing MIBG imaging for HF vary widely in age, body composition, and clinical background. Additionally, in Japan, the typical patient population undergoing MIBG imaging tends to be older, making a younger reference population less practical for routine use, as also observed in the JSNM Working Group’s Anger camera database. While population-specific databases (Jp- and It-NDBs) remain valid options, our analysis demonstrated that the combined JpIt-NDB closely aligns with each individual NDB and shows only minimal differences in defect scores. Thus, the combined database offers flexible, sensitive, and broadly applicable reference suitable for both clinical practice and multinational research.

## Diagnostic value of automated defect scores generated by JpIt-NDB

Traditionally, the HMR has been the primary parameter for assessing global sympathetic activity in ^123^I-MIBG cardiac scintigraphy. However, segmental defect scores derived from polar maps provide complementary insight into regional variations in sympathetic innervation. These scores are useful for evaluating myocardial damage and contractile dysfunction, which may not be evident from global HMR alone [13]. This approach is especially valuable in conditions where HMR is diffusely reduced, as in advanced HF [18].

Nevertheless, physiological profiles of lower uptake in inferior or apical regions need to be considered when calculating ^123^I-MIBG defect scores, as it can lead to overestimation [8–10, 39]. Consistent with prior findings, our study showed that visual defect scores were consistently higher than automated scores based on the JpIt-NDB, even when automated scores were zero, highlighting the challenges of distinguishing true abnormalities from physiological hypoactivity. This overestimation compromises diagnostic accuracy, particularly by reducing specificity, and is more pronounced in late images. Moreover, although mismatch scores between perfusion and sympathetic imaging have been applied, the latter has been based on visual comparisons or ratios (%) of regional counts [40, 41]. To minimize overestimation caused by non-specific reduction in the inferior wall, we recommend the use of automated scoring with appropriate NDBs. The HMR is reduced under various cardiac conditions that contribute to HF, including cardiomyopathy and acute myocardial infarction [42]. Consistent with these findings, we also found significantly decreased HMRs among patients with both CAD and DCM on early and late images. However, automated defect scores were unexpectedly similar between patients with DCM and controls and markedly higher in patients with CAD. Regional and global parameters also significantly differ between CAD and DCM [34]. That study found higher washout rates (WRs) in perfused segments than in damaged areas among patients with DCM, which contradicted what is typically associated with CAD. Furthermore, these rates were also significantly higher than in CAD, indicating the distinct pathophysiological mechanisms that underlie these conditions. A possible explanation for this is that neuronal loss in CAD typically accompanies myocardial damage and necrosis, with a clear demarcation between normal and affected areas. In contrast, DCM is characterized by more diffuse sympathetic nervous system dysfunction [43]. This hampers accurate assessments of defect extent in individual segments and potentially leads to underestimation. Therefore, we emphasized the importance of comprehensively evaluating global myocardial uptake (HMR) and regional derangements (early and late defect scores). The diagnostic effectiveness of this combination at the early and late stages of assessment was significantly increased compared with either method alone. This strategy compensates for the sensitivity limitations of these methods when applied individually, providing a more robust and reliable evaluation of myocardial innervation.

Despite extensive investigations into cardiac diseases using ^123^I-MIBG, detailed evaluations of combined HMR and defect scores remain limited, underscoring the need for further studies to optimize the diagnostic accuracy and improve clinical outcomes.

## Limitations

This study has several limitations. In addition to the HMR and defect scores, the WR is a crucial ^123^I-MIBG imaging parameter that reflects the neuronal integrity of sympathetic tone and adrenergic drive in the heart [44]. However, various calculation formulas among institutions introduce significant heterogeneity in WRs [45]. Specifically, the WRs were calculated using count-based planar imaging with an Anger camera in Japan, whereas in Italy, only HMRs were obtained using D-SPECT. D-SPECT does not retain raw count values in DICOM files, as it stores only proportional counts. Consequently, WR could not be calculated for the Italian cohort. Due to these limitations in standardization and inter-institutional comparisons, WR was excluded from the present analysis.

Furthermore, the limited number of normal persons used for validation, particularly within the Italian population, might have affected the diagnostic efficacy of automated defect scores derived from the JpIt-NDB. This limitation is primarily due to more stringent insurance regulations in Italy regarding reimbursement for cardiac ^123^I-MIBG imaging, which restricts access to this imaging modality among non-HF persons. Although the prognostic value of ^123^I-MIBG imaging has been established [42], this study did not assess that of the automated defect score from the JpIt-NDB. This was hampered by the fact that only 4 of 43 patients in our cohort died, which was insufficient for meaningful subgroup analysis. Future studies should address these limitations, strengthen our findings, and extend the observation period.

## Conclusions

We developed three NDBs for ^123^I-MIBG D-SPECT based on Japanese, Italian, and combined cohorts. The diagnostic performance of the combined-NDB for HF closely aligned with that of the population-specific databases (Jp- and It-NDBs), supporting its applicability in multinational research. Furthermore, integrating the HMR with NDB automated defect scores significantly enhanced the precision of cardiac assessments. These results not only validated the ^123^I-MIBG NDBs, but also provide promising avenues for future investigation into cardiac innervation imaging.

## Data Availability

The datasets generated and/or analyzed in the current study are not publicly available due to approval of the Ethics Committee, but are available from the corresponding author on reasonable request.

## Abbreviations

AUC: Area under the receiver operating characteristic curve
CAD: Coronary artery disease
DCM: Dilated cardiomyopathy
HMR: Heart-to-mediastinum ratio
JpIt: Japan and Italy
LVEF: Left ventricular ejection fraction
MIBG: Meta-iodo-benzyl-guanidine
NDB: Normal database
ROC: Receiver operating characteristic curve
SES: Summed early score
SLS: Summed late score
